# Presence of dogs and proximity to a wildlife reserve increase household level risk of tungiasis in Kwale, Kenya

**DOI:** 10.1186/s41182-021-00338-8

**Published:** 2021-07-05

**Authors:** Peter S Larson, Masanobu Ono, Mwatasa Changoma, Kensuke Goto, Satoshi Kaneko, Kazuhiko Moji, Noboru Minakawa

**Affiliations:** 1 Nagasaki University Institute of Tropical Medicine-Kenya Medical Research Institute (NUITM-KEMRI) project, Kenya, Nagasaki, Nagasaki, Japan; 2 University of Michigan School of Natural Resources and Environment, Ann Arbor, MI, USA; 3 Chemi Chemi, Kwale, Kenya; 4 Division of Health and Safety Sciences Education, Osaka Kyoiku University, Osaka, Japan; 5 Nagasaki University School of Tropical Medicine and Global Health (TMGH), Nagasaki, Japan

**Keywords:** Tungiasis, Zoonosis, Wildlife, Survey

## Abstract

**Introduction:**

Tungiasis is a ectopic skin disease caused by some species of fleas in the *Tunga* genus, most notably *T. penetrans*. The disease afflicts poor and marginalized communities in developing countries. Transmission of tungiasis comprises a complex web of factors including domesticated animals and wildlife. This research explores animal and environmental risk factors for tungiasis in an area adjacent to a wildlife reserve in Kwale, Kenya.

**Methods:**

A two-stage complex sampling strategy was used. Households were selected from three areas in and around Kwale Town, Kenya, an area close to the Kenyan Coast. Households were listed as positive if at least one member had tungiasis. Each household was administered a questionnaire regarding tungiasis behaviors, domesticated animal assets, and wild animal species that frequent the peridomiciliary area. Associations of household tungiasis were tests with household and environmental variables using regression methods.

**Results:**

The study included 319 households. Of these, 41 (12.85%) were found to have at least one person who had signs of tungiasis. There were 295 (92.48%) households that possessed at least one species of domesticated animal. It was reported that wildlife regularly come into the vicinity of the home 90.59% of households. Presence of dogs around the home (OR 3.85; 95% CI 1.84; 8.11) and proximity to the park were associated with increased risk for tungiasis infestation in humans in a multivariate regression model.

**Conclusions:**

Human tungiasis is a complex disease associated with domesticated and wild animals. Canines in particular appear to be important determinants of household level risk.

## Introduction

*Tunga penetrans*, known variously as the “jigger flea,” the “chigoe flea”, “nigua,” or “pico,” is a zoonotic ectoparasite common to developing countries [1, 2]. After attaching itself to the human host, the female flea burrows into the skin, primarily on the feet and lower extremities, causing the condition known as tungiasis [3]. Tungiasis is associated with a wide range of outcomes including itching, pain, secondary bacterial infections, severely impaired walking ability, and social marginalization [4–6]. Gangrene, necrosis, and bacterial superinfections are common complications of tungiasis which can result in loss of limbs or death [2, 7].

Females and males can attach to mammalian hosts. The unfertilized female burrows head first into the skin, leaving the last three abdominal segments, the so-called abdominal cone, protruding above the skin [8, 9]. It is through the abdominal cone that the female sand flea is fertilized, expels eggs, excretes fluid and fecal matter, and breathes [9].

Copulation occurs on the host. The flea subsists on blood from capillaries in the dermis. Within 2 weeks, she will increase in volume by more than 1000 times and produce eggs which will be expelled from the penetration site [9, 10]. An immune reaction results in swelling and itching around the embedded flea [10, 11]. Expelled eggs are deposited in the soil or on the floor. Larvae emerge and feed on organic material in the soil or in crevices and holes within the home [1]. Adults emerging from the pupal state will then attach to and penetrate the skin of humans when they walk barefoot, sit, or sleep on contaminated surfaces [12]. *T. penetrans* can complete its life cycle fully within the home, but animals and humans can also bring eggs into the home. Although infestations occur mainly on the feet, ectopic infestations can occur on the hands, elbows, knees, palpebral and tongue [3, 13–17].

Persistent scratching and the use of non-sterilized items such as thorns and sticks may induce secondary bacterial infections, often involving multiple bacterial species. *Staphylococcus aureus*, *Clostridium spp.*, and enterobacteriaceae are pathogens that have been reported in cases arising from Brazil [18, 19]. Tetanus is a documented outcome of tungiasis, particularly in areas where vaccination rates are low [20, 21]. Sometimes, these secondary infections lead to gangrene resulting in loss of extremities [12, 22]. Though the most common treatment method for mild infestations in a formal health facility is excision and administration of antibiotics depending on severity, several chemical and medicinal treatments have been suggested for severe and complicated infestations, with varying levels of success [9, 23–27]. Plant-based repellents have also been suggested to prevent new cases [28, 29]. Tungiasis receives little coverage in public health research and, given its propensity to afflict the poorest of the poor, is considered a classic case of the neglected tropical disease (NTD). It is expected to increase in public health importance with climate change [30–32].

Tungiasis is a zoonosis [33–35] and has been found in numerous species of wild and domesticated animals including monkeys, anteaters, goats, elephants, and rats [36–38]. Pigs have been shown to be a major reservoir for *T. penetrans* in Uganda, Nigeria, Brazil, and other areas [31, 33, 39, 40]. Tungiasis is common in dogs in many areas around the world [41–45]. Tungiasis cases in humans have also been associated with the presence of specific animal species around the home [46] and seasonal patterns of tungiasis in humans and animals have been found to overlap [47].

It is suspected that the ecological determinants of tungiasis include a complex web of factors that include humans, domesticated animals, and wildlife [1]. This research aims to explore possible domesticated and wild animal-related determinants of tungiasis using a cross sectional study of households in a rural community bordering a wildlife reserve in Kwale, Kenya. We test for associations of tungiasis with individual, household and environmental factors, such as the possession of various species of domesticated animals and presence of wildlife species around the home.

## Methods

### Ethical considerations

All participants gave written consent for administration of questionnaires and Clinical inspection. Consent to children’s participation was given by parents.

### Study area

**Fig. 1:**
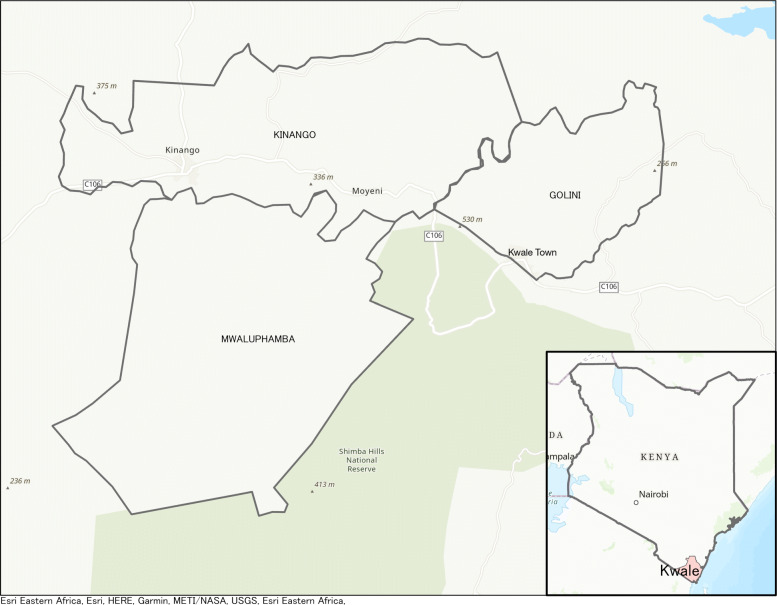
Location of study area. The three regions of Golini, Mwalaphamba, and Kinango and the area that comprises the Shimba Hills Wildlife Preserve are shown. Kwale Town is the center of the Kwale County Government and the most urbanized center of the region

This study was conducted as a part of an ongoing project for the Kwale Health Demographic Surveillance System (KHDSS) by the Nagasaki University Institute of Tropical Medicine, Kenya Medical Research Institute (NUITM-KEMRI) [48]. The KHDSS is based in Kwale County, Kenya, an area located along the coast of the Indian ocean, approximately 27 km from Mombasa. The study area is located between 4^∘ 17’N and 4^∘ 5’ S and between 39^∘ 15’W and 39^∘ 29’ E (Fig 1). The KHDSS study site covers a geographic area of approximately 443.2 km ^2^ comprising ~12,000 households and ~50,000 residents as of March 2017.

### Study design and population

Assuming a 5% margin of error and a 95% confidence level, the minimum sample size required to detect a population proportion of 5% was 73 households. We based this sample size calculation on results of a previous, unpublished survey of approximately 10,000 households on the same population in the same region. That study did not collect information on the same risk factors as this study.

Households were sampled from the KHDSS using a two-stage cluster sampling design. The KHDSS area is organized using a grid based geographical address system that assigns a unique ID to each house. The area is divided into 700 m × 700 m square meter grid cells, which are themselves subdivided into 100m × 100m sub-grids so that each grid cell has 49 sub-grids. Within each sub-grid, households are assigned a unique number. In the KHDSS, there are 652 grid cells divided between three regions: Golini 84, Kinango 228, and Mwaluphamba 340. We randomly chose 15 grid cells within each of the three KHDSS areas. Seven homes within each grid cell were then randomly selected from all homes in the cell, producing a total of 315 homes. To account for possible refusals or persons being away from the home at the time of the survey, and because time allowed, we purposely randomly selected 8 households from 10 randomly chosen grid cells to produce a total of 325 selected homes.

### Clinical inspection

Tungiasis cases were identified based on self report. If a person self reported having tungiasis, or a representative identified a family member (e.g., a child) as being infected, the person was asked to show lesions to field staff for confirmation. Homes were recorded as having tungiasis if there was at least one self-reported and confirmed tungiasis case in the home.

### Collection of epidemiological data

A full accounting of domesticated animals was performed during the household visit. Animals were visually inspected by lay field staff for signs of tungiasis with the assistance of household heads. Tungiasis status was measured at the herd level. An animal species was considered positive when one or more animals of that species were infested. No biological samples were collected from animals and survey workers had no physical contact with animals at any time.

A survey instrument was created and administered to household representatives. The survey included questions on topics such as species of animals raised in the household, watering behaviors, contact with wildlife, entry into the wildlife reserve, and observed wildlife species within the vicinity of the household. The questionnaire was prepared in English and translated into Swahili and Duruma. Responses were recorded with a digital tablet device (Samsung Galaxy tab A SM-T355) using Kimetrica survey data collection software [49]. Data was uploaded to a central server on a semi-daily basis. The variables age, sex, total number of family members, and latitude/longitude coordinates of the household were obtained from the KHDSS database. Socioeconomic status (SES) was measured using a multi-correspondence analysis (MCA)-based composite of household assets collected during a previous KHDSS survey round. Continuous SES measures were divided into quantiles following a procedure common to studies of SES in developing countries [50, 51].

One goal of this research was to test for association between environmental factors and household tungiasis. Previous, unpublished analyses of data collected in this region indicated that distance to the wildlife reserve might have some association with individual tungiasis risk. Examining wildlife species in the park for tungiasis infestation was far outside the scope of this research so the researchers used distance to the park as a proxy for wildlife movement in and out of the reserve. Latitude/longitude locations were used to test associations of household locations with proximity to the Shimba Hills Wildlife Reserve along with other environmental factors such as elevation, distance the nearest water source and distance to the nearest health facility. Distances from households to water sources and the wildlife park were measured in meters using Euclidean (“as the crow flies”) distances to the closest point on the closest polygon or line feature using the gdistance package in R [52]. A shapefile of the boundaries of the Shimba Hills Wildlife Reserve was obtained from the World Resources Institute [53]. Elevation and water sources such as rivers and lakes were obtained from DIVA-GIS [54]. Locations of health facilities were obtained from the Kenya Ministry of Health [55].

### Statistical analysis

Basic descriptive statistics were produced for the full data set. Univariate logistic regression models were use to identify risk factors with household level presence or absence of human and livestock tungiasis. As the analysis was at the household level, no attempt was made to test for associations of risk factors with individuals. To create a multivariate model of household tungiasis, a backwards selection procedure was used. A full model was created including all available variables. Variables that had estimates that had the highest p values were successively removed from the model until a best model was found based on Akaike’s Information Criterion (AIC) [56].

## Results

### Demographic characteristics of surveyed households

**Fig. 2:**
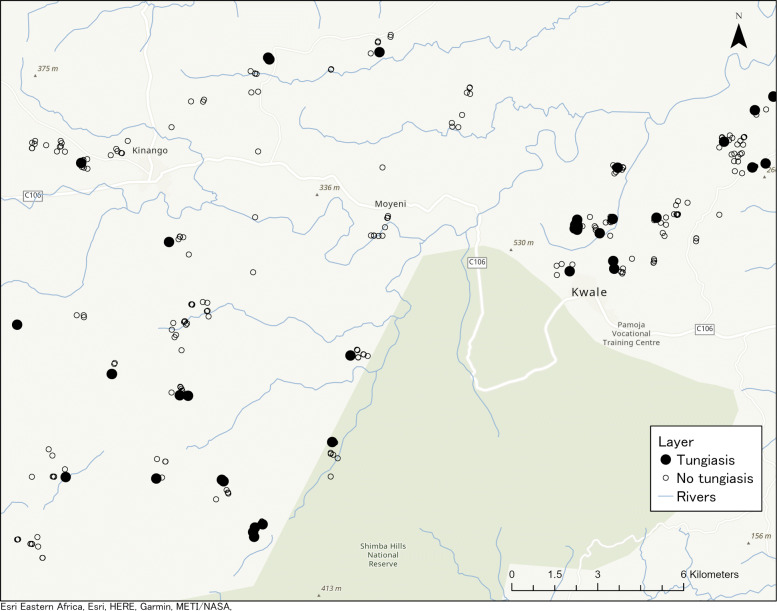
Locations of surveyed households and Shimba Hills Wildlife Reserve

Data was collected during February and March of 2017. Locations of sampled households in proximity to the Shimba Hills Wildlife Reserve are shown in Fig. 2. Data on 6 of the 325 households were lost. The final data set consisted of 319 households (Golini 117, Kinango 90 and Mwaluphamba 112) comprising approximately 1700 individuals.

Approximately 13% (41) of households had at least one member who was infected with tungiasis with household level tungiasis prevalence varying between the three regions (though this difference was not statistically significant). Males comprised 41.44% of people in each household. The median age of household members was 17 (range 1–83). The median number of household members was 5 (range 1–17). Selected households did not differ significantly between the three regions for most demographic measures. SES, however, differed between the three regions. Mwaluphamba, a very rural area, had a large percentage of very poor households and Golini, where Kwale Town is located, had a higher overall SES in this sample. See Table 1 for demographic results.

**Table 1:** Demographic comparison of sampled regions

	[ALL]	Golini	Kinango	Mwaluphamba	p.overall
	N = 308	N = 117	N = 79	N = 112	
Tungiasis	41 (12.9%)	19 (16.2%)	6 (6.67%)	16 (14.3%)	0.113
Number of people in home	5.00 [3.00; 7.00]	5.00 [3.00; 7.00]	5.00 [3.00; 6.50]	6.00 [3.00; 8.00]	0.092
Median age of people in household	20.1 (14.2)	21.4 (14.5)	20.6 (13.4)	18.4 (14.4)	0.259
Fraction male	41.4 (25.2)	41.5 (25.9)	44.6 (25.5)	39.2 (24.3)	0.361
SES categories:					¡0.001
Most poor	48 (20.3%)	2 (2.15%)	12 (23.1%)	34 (37.4%)	
4	45 (19.1%)	4 (4.30%)	17 (32.7%)	24 (26.4%)	
3	43 (18.2%)	14 (15.1%)	12 (23.1%)	17 (18.7%)	
2	60 (25.4%)	43 (46.2%)	5 (9.62%)	12 (13.2%)	
Least poor	40 (16.9%)	30 (32.3%)	6 (11.5%)	4 (4.40%)	

### Domesticated animals and tungiasis infestation

It was found that 295/319 (92.48%) households raised at least one species of domesticated animal. Among these, 61.44% of all households possessed at least one goat, followed by cows (31.03%) and sheep (9.40%). Most households (83.70%) reported raising at least one species of bird (e.g. chickens, ducks, turkeys).

Visual inspection by staff that did not possess specialized training in veterinary science or practice indicated that infestation varied among animal species. Infestation prevalence was assessed at the species level. Each species was recorded as “infested” if at least one animal examined showed signs of tungiasis infestation. Each percentage presented is the fraction of infected herd among the total number of homes that possessed that species. Goats were the most commonly infested species (54.08%), followed by cows (62.63%) and sheep (53.33%). Chickens and other birds were found to be infested (55.81%). Household cats and dogs were also found to be infested with tungiasis (26.87% and 56.9%, respectively). See Table 2 for full results.

**Table 2:** Number of herds examined and infested by Tunga sp. Examinations were performed visually by lay staff members with assistance of household heads

Animal_species	Number of herds n (%)	Number of infested herds n (%)
Cows	99 (31.03%)	62 (62.63%)
Goats	196 (61.44%)	104 (53.06%)
Birds	267 (83.7%)	147 (55.06%)
Cats	134 (42.01%)	36 (26.87%)
Dogs	58 (18.18%)	33 (56.9%)

Several animal owners reported removing embedded fleas from their animals (156/194; 70.61%) and themselves (221/313; 70.61%.) Implements used included needles (212/220; 96.36%) and oils or topical herbal or chemical treatments (8/220; 3.64%). (Results not shown in tables)

### Contact of household domesticated animals with wildlife

Some (37/319; 11.59%) households reported one or more species of animals in their possession had regular contact with wildlife. The most commonly cited location of livestock and wildlife contact was grazing areas (86.49%). Most households reported that wildlife come into the vicinity of the home (90.60%). They reported that household animals come into contact with a variety of wildlife species including baboons, buffalo, monkeys, snakes, leopards, warthogs, antelope, and others.

### Determinants of human tungiasis at the household level

We did not find an association between the number of people in the home, the median age of household members, SES, building materials, and water source with the odds of having at least one tungiasis case in the home. We also did not find an association of household tungiasis with the possession of most species of domesticated animals or with wildlife species regularly seen with within the vicinity of the home. We did, however, find that dogs were associated with increased odds of having tungiasis (OR = 2.24; 95% CI = 1.12–4.45). See Table 3 for full results.

**Table 3:** Univariate associations of household level human tungiasis (presence/absence of tungiasis case) with household, animal and environmental variables

	[ALL]	No	Yes	OR	p.ratio
	N = 319	N = 278	N = 41		
Number of people in home	5.00 [3.00; 7.00]	5.00 [3.00; 7.00]	5.00 [2.00; 7.00]	0.90 [0.80; 1.02]	0.091
Median age of people in household	20.1 (14.2)	19.9 (13.4)	21.4 (19.1)	1.01 [0.98; 1.03]	0.558
Fraction male	41.4 (25.2)	42.0 (25.0)	37.2 (26.9)	0.99 [0.98; 1.01]	0.274
SES categories:					
Most poor	48 (20.3%)	43 (20.9%)	5 (16.7%)	Ref.	Ref.
4	45 (19.1%)	35 (17.0%)	10 (33.3%)	2.40 [0.76; 8.55]	0.136
3	43 (18.2%)	38 (18.4%)	5 (16.7%)	1.13 [0.28; 4.51]	0.859
2	60 (25.4%)	53 (25.7%)	7 (23.3%)	1.13 [0.33; 4.16]	0.851
Least poor	40 (16.9%)	37 (18.0%)	3 (10.0%)	0.71 [0.13; 3.23]	0.664
**Domesticated animals**					
Cows	0.31 (0.46)	0.31 (0.46)	0.34 (0.48)	1.18 [0.59; 2.36]	0.645
Goats	0.61 (0.49)	0.62 (0.49)	0.59 (0.50)	0.87 [0.45; 1.69]	0.682
Birds	0.84 (0.37)	0.83 (0.38)	0.88 (0.33)	1.46 [0.55; 3.93]	0.448
Cats	0.42 (0.49)	0.42 (0.49)	0.41 (0.50)	0.97 [0.50; 1.90]	0.940
Dogs	0.30 (0.46)	0.28 (0.45)	0.46 (0.51)	2.24 [1.12; 4.45]	0.022
**Wildlife species**					
Elephants	0.15 (0.35)	0.14 (0.35)	0.18 (0.39)	1.34 [0.55; 3.27]	0.523
Warthogs	0.35 (0.48)	0.36 (0.48)	0.26 (0.44)	0.61 [0.28; 1.31]	0.203
Rabbits	0.02 (0.13)	0.02 (0.14)	0.00 (0.00)	0.00 [0.00;.]	0.989
Baboons	0.33 (0.47)	0.33 (0.47)	0.31 (0.47)	0.91 [0.44; 1.88]	0.789
Monkeys	0.01 (0.12)	0.01 (0.11)	0.03 (0.16)	2.16 [0.22; 21.3]	0.510
Hyena	0.07 (0.25)	0.06 (0.25)	0.08 (0.27)	1.21 [0.34; 4.37]	0.767
Wild cats	0.61 (0.49)	0.61 (0.49)	0.62 (0.49)	1.02 [0.51; 2.04]	0.953
Mongoose	0.01 (0.12)	0.01 (0.11)	0.03 (0.16)	2.16 [0.22; 21.3]	0.510
Buffalo	0.01 (0.10)	0.01 (0.11)	0.00 (0.00)	0.00 [0.00;.]	0.987
Zebra	0.00 (0.06)	0.00 (0.06)	0.00 (0.00)	0.00 [0.00;.]	0.989
**Household and environmental factors**					
Distance to health facility	3.37 (1.72)	3.37 (1.71)	3.43 (1.82)	1.02 [0.85; 1.24]	0.816
Distance the nearest river or stream	3.06 (2.00)	2.99 (1.95)	3.50 (2.24)	1.13 [0.96; 1.31]	0.132
Distance to wildlife reserve	6.48 (3.74)	6.63 (3.75)	5.43 (3.60)	0.91 [0.82; 1.00]	0.057
Elevation	227 (81.9)	223 (77.7)	253 (103)	1.00 [1.00; 1.01]	0.032
Wall materials:					
Brick/block	16 (6.78%)	13 (6.31%)	3 (10.0%)	Ref.	Ref.
Mud/cement	13 (5.51%)	11 (5.34%)	2 (6.67%)	0.81 [0.08; 6.27]	0.840
Stone	6 (2.54%)	5 (2.43%)	1 (3.33%)	0.93 [0.03; 10.4]	0.957
Wood/mud	201 (85.2%)	177 (85.9%)	24 (80.0%)	0.57 [0.17; 2.75]	0.439
Water_source:					
Open well	26 (11.0%)	21 (10.2%)	5 (16.7%)	Ref.	Ref.
Piped	117 (49.6%)	106 (51.5%)	11 (36.7%)	0.43 [0.14; 1.53]	0.182
Pond/dam	45 (19.1%)	39 (18.9%)	6 (20.0%)	0.65 [0.17; 2.57]	0.525
Stream/river	48 (20.3%)	40 (19.4%)	8 (26.7%)	0.83 [0.24; 3.15]	0.780

While the association was weak (p =.091), the odds of having tungiasis was lower for each additional person in the home (OR =.90; 95% CI =.80–1.02). Increased distance to the wildlife reserve (in kms) was also weakly (p =.057) associated with a decreased odds of having a tungiasis case in the home (OR =.91; 95% CI =.82–1.00).

### Multivariate model of household tungiasis

We created a reduced multivariate model from a full model including all of the variables included in Table 3. Variables were successively removed based on significance until an optimal model (using Akaike’s Information Criterion) was reached. Though the model had poor predictive power, it suggested that increased numbers of people in the home was associated with a reduced odds of a household member having tungiasis (OR = 0.41; 95% CI = 0.16–1.02). The presence of dogs around the home significantly increased the odds of a household having a positive case of tungiasis (OR = 3.85; 95% CI = 1.84–8.11). The reverse selection procedure left “mongoose” in the model. The association of mongoose with household tungiasis was positive and significant, but the confidence interval was extremely wide (OR = 11.07; 95% CI = 0.49–109.50). We note that “mongoose” (Swahili : nguchiro) might mean different things to different people including small mammals. None of the livestock variables remained. Finally, increasing distance in kilometers from the wildlife park was associated with a decreased odds of having a positive case of tungiasis in the home (OR = 0.86; 95% CI = 0.76—0.95). See Table 4 for full results

**Table 4:** Multivariate model of household tungiasis

	OR (95% CI)	p.ratio
(Intercept)	0.41 [0.16–1.02]	.059
Number of people in home	0.89 [0.78–1.01]	.072
Dogs around the home	3.85 [1.84–8.11]	< 0.001
Mongoose around the home	11.07 [0.49–109.50]	0.055
Distance to wildlife reserve	0.86 [0.76–0.95]	0.005
Observations	305	
Marginal R2/conditional R2	0.089 / NA	

## Discussion

We have shown that the presence of dogs and close proximity to a wildlife reserve are possible determinants of human tungiasis in this region of Kenya using a small, two stage, complex sampling-based study and a multivariate regression model. These results might suggest that dogs play an intermediate role between wildlife tungiasis and human tungiasis. The wider range of movement of dogs might put them at risk for infestation in wildlife rich areas. Access to areas where humans live might then allow them to bring eggs in and around the home, where they mature into adult fleas and proceed to infest humans and human living areas. However, this link remains to be shown empirically and our results should not be considered as evidence that a definitive connection between wildlife and human tungiasis exists.

Dogs have been implicated as risk factors for human tungiasis in other studies [41, 44, 57–62]. Increased numbers of lesions in dogs and cats are associated with more human infestations in Brazil. In that study, only dogs and cats, known to be reservoirs for a number of human parasites [63], were found to harbor the flea [34]. Another study in Brazil found that major risk factors for canine tungiasis were semi-restriction and sandy soils in household compounds [64]. Interventions which target dogs have been offered as potential solutions to controlling transmission in poor communities [45].

Our results from the univariate and multivariate models suggest that risk for tungiasis at the household level might be graded along distance to the reserve. Unpublished, previously collected data from the same region suggest that individual cases of human tungiasis are concentrated in the areas close to the park borders. Increased risk for tungiasis in close proximity to the reserve might suggest that wildlife act as reservoirs for *T. penetrans*. The relationship, however, could be ecological, with wildlife rich areas simply having a confounding relationship with human tungiasis. Risk for tungiasis in the home might be influenced by soil type, water, or specific human activities. More work through larger and more geographically expansive surveys is needed to better understand the complex ecology of *T. penetrans* and the role of diverse wildlife and other environmental factors in creating conditions suitable for transmission.

There were many limitations to this study. First, the self-reported nature of case identification might have introduced reporting biases into the analysis. This study relied on household heads to report the case status of themselves and other household members so that many cases, particularly among children, might have been missed. Next, the diagnostic ability of field workers may have been insufficient to properly identify cases. Survey workers were given basic training in case identification, and it was assumed that previous experience in conducting other similar surveys on tungiasis would have sufficiently prepared them for case identification in this study. This assumption turned out to be incorrect. For example, we attempted to use the Fortaleza classification system [10] to grade lesions but found that field workers had difficulty applying it. As the results were uninformative, they were excluded from the analysis. This experience demonstrated that lay field workers had trouble identifying tungiasis lesions. However, cases were confirmed visually, so it is unlikely that prevalence was over-estimated.

Future field studies of tungiasis in this region that include clinical diagnoses should be conducted in cooperation with trained medical staff. Had a clinical officer or health worker inspected and diagnosed all household members, household level prevalence may have been found to be much higher. Moreover, dust and dirt on animals feet might obscure infestations so that infections in animals might also be an underestimate. If studies are to use lay field staff to identify cases in animals or humans, great effort should be made to train and monitor their work. Recorded results should be validated if precise data are to be collected to assess prevalence and test for associations with risk factors.

We found that the two stage cluster sampling approach was suitable for this area and saved considerable time and effort in data collection. We found that, despite the problems with case identification, our approach sufficiently estimated community tungiasis prevalence when validated against previous, more comprehensive surveys. We also found that our approach of selecting clusters did not introduce spatial biases into the data or miss areas that might be of high risk for tungiasis. We would suggest that future studies of tungiasis in this area utilize such an approach.

Tungiasis varies significantly between rainy and dry seasons [65]; our study, conducted in the dry season, may have been appropriate to capture cases as they might occur over the full yearly cycle. The increased efficiency of data collection introduced by the two-stage cluster design might allow for longitudinal studies that provide more informative results on temporal and seasonal patterns of disease incidence. Regular data collection might allow us to better characterize seasons of disease incidence and also provide an added benefit of allowing regular data collection on tungiasis in domesticated animals and associations with wildlife movements. While this study was performed in Kenya and *T. penetrans* is the only species present on the African subcontinent, future, more geographically inclusive studies should attempt to identify the species of parasite and test for differences in prevalence and risk factors.

The most important limitation of this study is the complete lack of data on tungiasis prevalence in wildlife species. Examinations of wild animals for signs of tungiasis was far outside the scope of this small study. Future studies might partner with the Kenya Wildlife Service or park authorities to examine wild animals, living or dead, for signs of tungiasis lesions. This could provide information on which species are infested with tungiasis. Spatial analyses might also provide information on how animal movements might be associated with the geographic distribution of tungiasis risk. Without information on tungiasis prevalence in wildlife, we can only speculate on the true associations between proximity to the wildlife preserve and tungiasis risk in humans. The association we found between household tungiasis and proximity to the park in the multivariate model might be merely an ecological association. Other environmental or social variables might be more important in determining risk. Regardless, future research should take advantage of the opportunities that this region offers to further examine the complexities of the ecology of *T. penetrans* and tungiasis transmission.

## Conclusions

Presence of dogs is a strong determinant of household tungiasis infestation. Small, wild mammals may also contribute to tungiasis risk but this relationship needs to be tested empirically. Close proximity to areas of abundant wildlife might also be a determinant of increased risk but the reasons for this association deserve more investigation. These results might suggest that the broader ecology of *T. penetrans* as it impacts human health is a complicated web involving multiple animal species.

## Data Availability

Data and materials are available upon request.

## Abbreviations

AIC: Akaike’s Information Criterion
CI: Confidence interval
IRB: Institutional review board
KEMRI: Kenya Medical Research Institute
KHDSS: Kwale Health Demographic Surveillance System
NUITM: Nagasaki University Institute for Tropical Medicine
OR: Odds ratio
SES: Socioeconomic status
